# A Case of Estrogen Dermatitis Successfully Treated With Spironolactone

**DOI:** 10.7759/cureus.100857

**Published:** 2026-01-05

**Authors:** Tulika Singhal, Hailey Olds, Aunna Pourang, Geoffrey A Potts

**Affiliations:** 1 Medicine, Oakland University William Beaumont School of Medicine, Rochester, USA; 2 Department of Dermatology, Wayne State University School of Medicine, Detroit, USA

**Keywords:** anti-androgen therapy, estrogen dermatitis, polycystic ovary syndrome (pcos), spironolactone, spironolactone therapy

## Abstract

Estrogen dermatitis is a rare dermatologic condition characterized by a rash that worsens during periods of rising estrogen and improves when estrogen levels fall during menstruation. Because of variability in morphology and histopathology, it may mimic and/or overlap with other inflammatory skin disorders. Here, we describe a case of suspected estrogen dermatitis associated with polycystic ovarian syndrome (PCOS), hidradenitis suppurativa, and acne. The patient noted symptom improvement during her menstrual cycle. Clinical presentation and histopathological findings were non-specific. Because of a history of migraines with aura, the patient was not a candidate for combined oral contraceptives. She was started on spironolactone, which led to complete resolution of the rash. This case highlights the potential of anti-androgen therapy, such as spironolactone, as a promising treatment option for estrogen-sensitive skin conditions, particularly in patients who have contraindications for estrogen-based therapies.

## Introduction

Estrogen dermatitis is a rare skin condition characterized by a rash that worsens during periods of rising estrogen and improves when estrogen levels fall during menstruation [1]. The exact mechanism is not fully understood, but it is thought that dendritic cells exhibit a hypersensitivity response to estrogen, resulting in estrogen-related skin inflammation [2]. This condition may manifest as urticaria, red papules, or eczematous patches. Because of this variability in morphology, it may mimic other inflammatory skin disorders and overlap with their clinical presentation [1,2]. Although immunohistochemical staining for estrogen receptors has been explored, no definitive diagnostic test exists. Diagnosis remains clinical, based on the patient's symptoms, medical history, and correlation with the menstrual cycle, and it is largely a diagnosis of exclusion. Tamoxifen may be effective in treating this condition, but it is not without side effects [2]. In this case report, we describe a patient with suspected estrogen dermatitis whose symptoms improved with spironolactone, a commonly used hormone-related medication, which may be a promising treatment option for this skin condition.

## Case presentation

**Figure 1:**
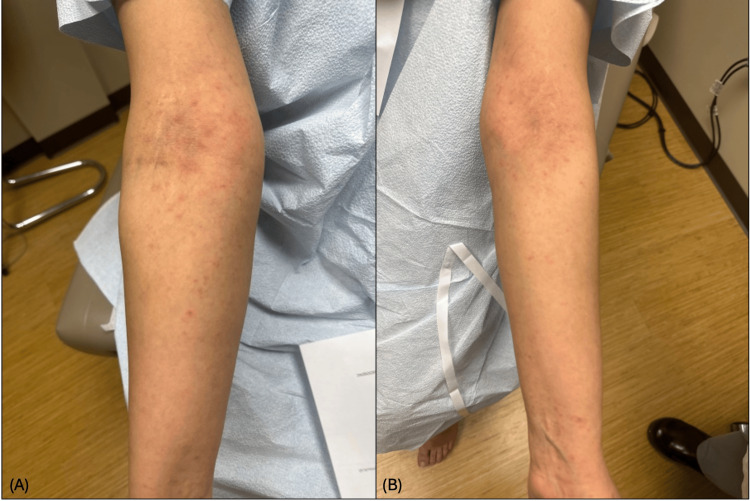
Erythematous papules on (A) the right antecubital fossa, arm, and volar forearm and (B) the left antecubital fossa, arm, and volar forearm

**Figure 2:**
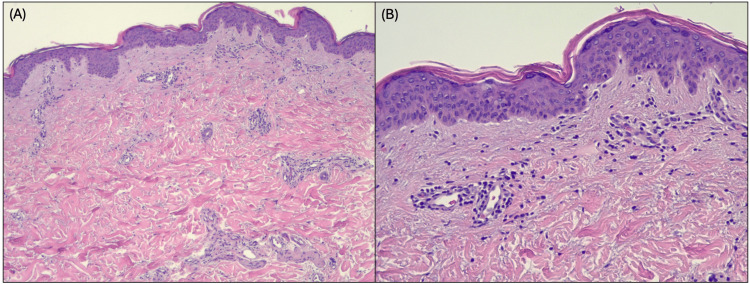
Histopathological examination under (A) 4x and (B) 40x magnification, demonstrating a sparse perivascular lymphocytic infiltrate and mild spongiosis

A 37-year-old female with a history of polycystic ovarian syndrome (PCOS), hidradenitis suppurativa, and acne presented with a pruritic rash affecting her arms, legs, and trunk. The rash had been present on and off for years. She noted symptom improvement during her menstrual cycle, and worsening the week afterwards. On examination, erythematous papules were noted on her arms, dorsal hands, back, chest, abdomen, and thighs (Figure 1). Biopsies from the left upper chest and lower back demonstrated subacute spongiotic dermatitis (Figure 2). Laboratory workup was negative for antinuclear antibodies and hepatitis B. Hormonal assays showed normal free testosterone, mildly elevated 17-hydroxyprogesterone (208), and an elevated luteinizing hormone to follicle-stimulating hormone ratio of 6.5, consistent with PCOS.

The patient tried and failed multiple therapies, including oral antihistamines, corticosteroids (oral and intramuscular), triamcinolone ointment, tacrolimus ointment, ruxolitinib cream, and methotrexate dosed at 12.5 mg weekly. Phototherapy was initially helpful but later caused worsening irritation and burning. Because of a history of migraines with aura, the patient was not a candidate for combined oral contraceptives. Tamoxifen was not tried because possible adverse reactions include additional rashes. She was started on spironolactone 100 mg daily, which was reduced to 50 mg after experiencing palpitations. The rash completely resolved on the lower dose within a few months.

## Discussion

The correlation between the patient's rash and her menstrual cycle suggested a hormone-sensitive skin condition. Estrogen dermatitis was favored over progesterone dermatitis because her symptoms improved during menstruation, when estrogen levels decline. In contrast, progesterone dermatitis typically flares during the luteal phase, when progesterone is elevated [2]. Her history of PCOS further supported the diagnosis, as hormonal imbalance in PCOS leads to elevated androgen levels that are converted to estrogen in peripheral tissues [3]. This relative estrogen excess may have contributed to her symptoms, given that estrogen dermatitis is believed to result from a hypersensitivity reaction to estrogen [2].

Due to the increased risk of stroke associated with migraines with aura, the patient was not a candidate for estrogen-based therapies such as oral contraceptive pills. Spironolactone, a potassium-sparing diuretic with anti-androgenic properties, was therefore trialed. This drug blocks androgen receptors and may reduce androgen biosynthesis [4]. Although spironolactone has been described in earlier literature as having weak estrogenic effects, mechanistic studies suggest that it does not exert clinically meaningful estrogen receptor agonism or increase estrogen synthesis at therapeutic concentrations. Receptor-binding studies have demonstrated that canrenone, the primary active metabolite of spironolactone, does not alter estradiol binding to estrogen receptors in human uterine cytosol, indicating a lack of direct estrogen receptor activation [5]. In addition, in vitro studies using human fetal liver cells have shown that spironolactone does not stimulate aromatase activity or enhance peripheral conversion of androgens to estrogen at physiologic doses [6]. In the context of PCOS, where excess androgen substrate may contribute to increased peripheral estrogen exposure, reduction of androgen signaling without increasing estrogen production may explain the observed clinical improvement in this patient. This case suggests that spironolactone may represent a possible therapeutic option for estrogen-sensitive dermatoses in select patients who cannot receive estrogen-based therapies. However, it is important to note that this patient had comorbid, untreated PCOS, which may have contributed to the improvement with spironolactone as well. 

## Conclusions

This is a rare case of presumed estrogen dermatitis successfully treated with spironolactone monotherapy. This case highlights the potential of anti-androgen therapy, such as spironolactone, as a treatment option for estrogen-sensitive skin conditions, particularly in patients who have contraindications for estrogen-based therapies and comorbid conditions. Anti-androgen therapy may be especially helpful in patients with comorbid PCOS, such as this patient. 
